# Delta-Tocotrienol Modulates Glutamine Dependence by Inhibiting ASCT2 and LAT1 Transporters in Non-Small Cell Lung Cancer (NSCLC) Cells: A Metabolomic Approach

**DOI:** 10.3390/metabo9030050

**Published:** 2019-03-13

**Authors:** Lichchavi Dhananjaya Rajasinghe, Melanie Hutchings, Smiti Vaid Gupta

**Affiliations:** Department of Nutrition and Food Science, Wayne State University, Detroit, MI 48202, USA; lichchavi.rajasinghe@wayne.edu (L.D.R.); dv2329@wayne.edu (M.H.)

**Keywords:** cancer, mTOR, vitamin E, SLC1A5, tocotrienols, apoptosis, cell growth, cell transporters, essential amino acids, ASCT2, glutaminolysis, alanine, glutathione, glutamate, lung, bio actives, nutraceuticals

## Abstract

The growth and development of non-small cell lung cancer (NSCLC) primarily depends on glutamine. Both glutamine and essential amino acids (EAAs) have been reported to upregulate mTOR in NSCLC, which is a bioenergetics sensor involved in the regulation of cell growth, cell survival, and protein synthesis. Seen as novel concepts in cancer development, ASCT2 and LAT transporters allow glutamine and EAAs to enter proliferating tumors as well as send a regulatory signal to mTOR. Blocking or downregulating these glutamine transporters in order to inhibit glutamine uptake would be an excellent therapeutic target for treatment of NSCLC. This study aimed to validate the metabolic dysregulation of glutamine and its derivatives in NSCLC using cellular 1H-NMR metabolomic approach while exploring the mechanism of delta-tocotrienol (δT) on glutamine transporters, and mTOR pathway. Cellular metabolomics analysis showed significant inhibition in the uptake of glutamine, its derivatives glutamate and glutathione, and some EAAs in both cell lines with δT treatment. Inhibition of glutamine transporters (ASCT2 and LAT1) and mTOR pathway proteins (P-mTOR and p-4EBP1) was evident in Western blot analysis in a dose-dependent manner. Our findings suggest that δT inhibits glutamine transporters, thus inhibiting glutamine uptake into proliferating cells, which results in the inhibition of cell proliferation and induction of apoptosis via downregulation of the mTOR pathway.

## 1. Introduction

Non-small cell lung cancer (NSCLC) presents itself as aggressive tumors arise from the airway epithelial cells (majority) and interior parts of the lungs [1]. It remains one of the leading causes of disease-related mortalities in the world. The current therapeutic options for NSCLC, which include surgery, radiotherapy, and chemotherapy [1], have slightly improved NSCLC survival rate at some developmental stages in both men and women. However, there has been a plateauing of the overall five-year survival rate, hovering ~12–18% between the years 1975 and 2011 [2]. Also, several studies report that there is a high probability of reoccurrence and development of resistance to drug therapies in NSCLC after treatment with chemotherapeutic agents, surgical resection, and radiation therapy [3]. This warrants efforts to identify novel therapeutic agents and targets for preventing and treating NSCLC.

Research in nutrition-based modulation against diseases has opened up new horizons in cancer prevention, contributing to drug discovery and development processes for numerous chronic diseases, including cancer [4,5]. Most bioactive agents extracted from plants show minimum cell cytotoxicity while simultaneously targeting multiple signaling pathways involved in cell growth, apoptosis, invasion, angiogenesis, and metastasis in cancer cells [6,7]. Tocotrienols (α, β, γ, and δ), isomers of vitamin E, are found in vegetable oils, including rice bran oil and palm oil, wheat germ, barley, annatto, and certain other types of seeds, nuts, and grains [8]. They exert biological effects including antiangiogenesis, antioxidant activities, and anticancer activities [9,10]. Our previous studies clearly demonstrated that delta-tocotrienol (δT) inhibits the proliferation and metastatic/invasion potential while concurrently inducing apoptosis in NSCLC cells, in a dose-dependent manner [11]. We also identified some of the probable molecular targets of δT treatments on NSCLC [11,12,13]. Therefore, δT is multitargeted and can be considered a valuable potential approach to further investigate for treatment of NSCLC.

Metabolomics, a novel, versatile, and comprehensive approach, can provide unbiased information about metabolite concentrations, altered signaling pathways, and their interactions. Most current cancer metabolomics studies focus on finding diagnostic biomarkers and understanding fundamental mechanisms in cancer [14]. Nonetheless, this approach could also be used effectively for identifying the efficacy of treatments [15]. The NSCLC metabolome is a potentially informative reflection of the impact of the disease and its dynamics which could lead to promising developments in cancer research, strongly geared toward the discovery of new biomarkers of disease onset, progression, and effects of treatment regimens. Given that cancer cells, including NSCLC, show aberrant energy metabolism [16,17], it is of interest to investigate the changes in energy metabolism in NSCLC cells upon δT treatment, utilizing the global advantage of the metabolomic approach [18].

Glutamine plays a role as an indirect energy source in NSCLC, which produces ATP through glutamine-driven oxidative phosphorylation [19]. Extra consumption of glutamine in tumors is used for generating metabolic precursors for uncontrolled cell proliferation. These precursors include elevated levels of nucleic acids, lipids, and proteins for cell proliferation [20], as well as increased GSH production for cell death resistance [21]. Current literature provides further evidence that glutamine in cancer facilitates exchange of EAAs (essential amino acids) with glutamine into proliferating cells via glutamine transporters, which induces mTOR (mammalian target of rapamycin) activation in NSCLC and other types of cancer [22,23]. Activated mTOR then promotes protein translation and cell growth via activation of its downstream genes such as S6k1 and 4EBP1 [24]. Alanine, serine, cysteine-preferring transporter 2 (ASCT2), also known as (SLC1A5), and bidirectional L-type amino acid transporter 1 (LAT1) are the two primary transporters for glutamine uptake [25,26]. LAT1 enables transport of the EAAs to improve cancer cell growth via mTOR-induced translations, and ASCT2 sustains the cytoplasmic amino acid pool to drive LAT1 function [27]. This collaboration of ASCT2 and LAT1 reduce apoptosis and enhance the energy production and cell growth via net delivery of glutamine inside the cell [27].

A recent study reported that A549 and H1229 lung cancer cells show glutamine dependency, and that deprivation of glutamine inhibits cell growth [28]. Decreases in glutamine uptake, cell cycle progression, and mTORC1 pathway after inhibition of ASCT2 functionality by chemicals or shRNA in vitro was observed in prostate and pancreatic cancer cell lines [29]. Also, inhibition of LAT1 using BCH (2-aminobicyclo-(2,2,1)-heptane-2-carboxylic acid) in H1395 lung cancer cell line reduced the cellular leucine uptake and consequently inhibited mTOR pathway activity, which finally reduced cell proliferation and viability [30]. Induction of apoptosis was also reported in hepatoma, hybridoma, leukemia, myeloma, and fibroblast cells after glutamine deprivation [31,32]. Our preliminary metabolomics studies showed that δT treatments inhibited glutamine levels in A549 and H1299 cells. Also, in our previous studies, induction of apoptosis and inhibition of cell growth was observed in A549 and H1299 cells in a dose-dependent manner after δT treatments [11,33,34,35,36]. Therefore, the aim of this study was to verify the metabolic dysregulation of glutamine and its derivatives upon δT treatment while investigating the effect of δT on the expression of glutamine transporters (ASCT2 and LAT1) and the mTOR pathway. 

## 2. Results

### 2.1. δT Changes Metabolite Profiles in A549 and H1299 Cells

**Figure 1:**
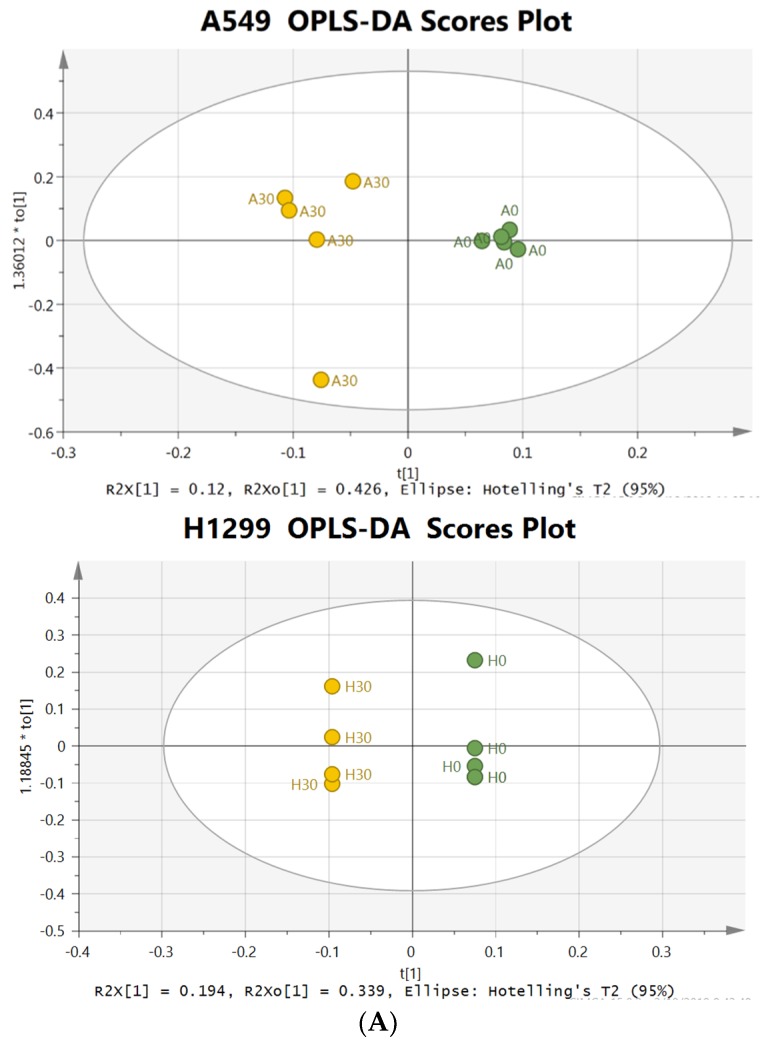

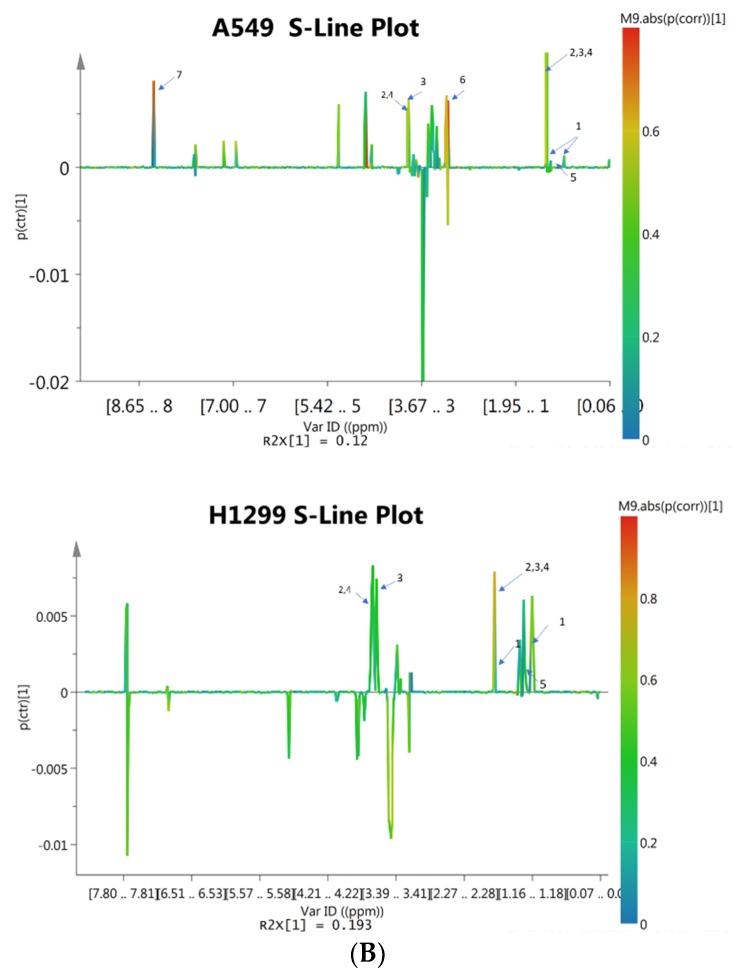
OPLS-DA analysis of metabolome of lung cancer cell lines after treating with/without δT for 72 h. (**A**) OPLS-DA Scores plot based on the cellular metabolic profiling of lung cancer cell lines, namely A549 (Top) and H1299 (Bottom); the 30 µM treatment (Yellow) and control (green) were generated using SIMCA+ software; the results indicated that cellular metabolic profiling of lung cancer cell lines was significantly changed after δT treatment for 72 h. (**B**) The S-Line plots of OPLS-DA analysis of A549 (top) and H1299 (Bottom) from treatment (30 µM) and control (0 µM) cells. The key metabolites that changed significantly are marked on the S-Line plot and include (1) leucine, (2) glutamine, (3) glutamate, (4) glutathione, (5) lactate, (6) taurine, and (7) formate.

To investigate the changes in metabolism and metabolites with δT intervention, supervised OPLS-DA analysis was performed using NMR spectral data acquired from intracellular cell lysate. The OPLS-DA score plot of cellular NMR metabolic profile resulting from 30 µM δT treated and control cells lines are shown in Figure 1A. The OPLS-DA score plot exhibited clear separation between control and treatment groups in A549 cells and H1299 cells with δT treatment; the high Q2 and R2 values indicate a considerable difference in the cellular metabolic profile of treated cells compared to control cells while validating the model that we used for OPLS-DA analysis.

To identify the metabolites represented in the NMR spectral regions (bins) that varied significantly between control and treatment groups, the corresponding loading S-Line plot from the OPLS-DA model was generated. Figure 1B shows a representative S-Line plot corresponding to the score plot of Figure 1. These bin numbers were further analyzed to identify the significant metabolites (using Chenomx) that contributed to the separation of the control and treatment groups seen in the OPLS-DA model. Based on the analysis of S-Line plot bin numbers, the key bin numbers responsible for the differences could be attributed to glutamine, glutamate and glutathione, and some amino acids in both cell lines. 

### 2.2. Quantification of Metabolites Reveals That δT Alters Glutamine Metabolism

Chenomx 7.6 Suite NMR software was used to probe the metabolome profiles in the treatment and control groups. 1H-NMR spectra provided information on over 45 metabolites (both cell lines), including amino acids, intermediates of the tricarboxylic acid cycle (TCA), energy molecules, and nucleic acid associated molecules (Table 1).

The table shows the detailed results including p-values, mean and standard deviation from the *t*-test for the groups (with or without 30 µM of δT treatment) tested. Among the metabolites that were significantly different in concentration in the δT treated vs. control cells, we identified several metabolites from the glutamine metabolism and related pathways that were significantly decreased (*p* < 0.05) in the treatment group as compared to controls. In addition, we found that metabolites such as leucine and some essential amino acids had significantly lower concentrations in both cell lines after δT treatment. These essential amino acids include isoleucine, leucine, lysine, methionine, and tryptophan. Moreover, the metabolites related to cell proliferation such as 2-oxoglutarate, citrate, succinate, malate, aspartame, ATP, ADP, NADPH, and uracil significantly decreased (*p* < 0.05) in the treatment group as compared to controls (Table 1). 

**Figure 2:**
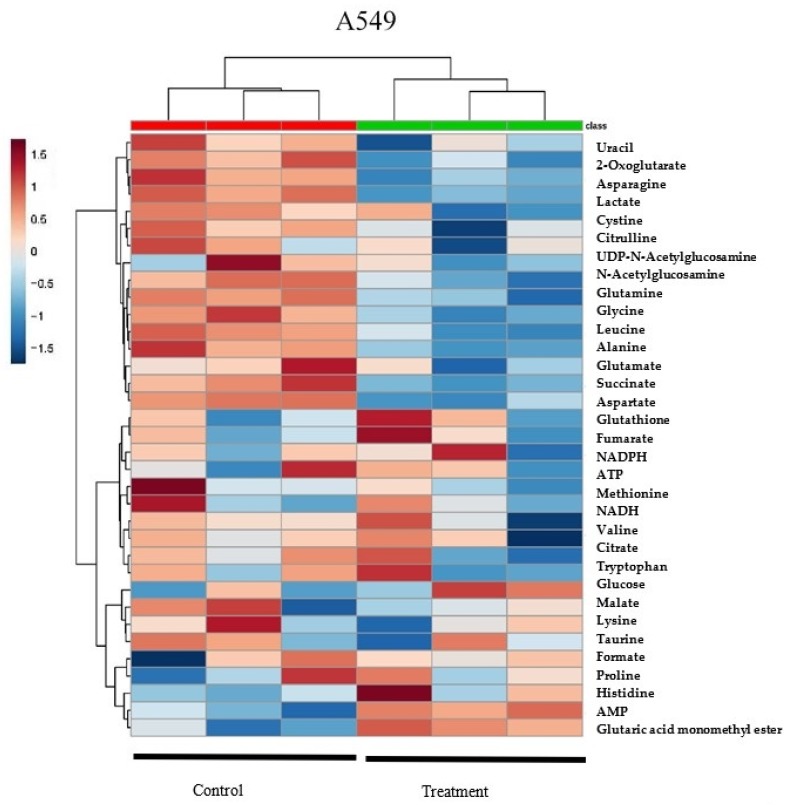

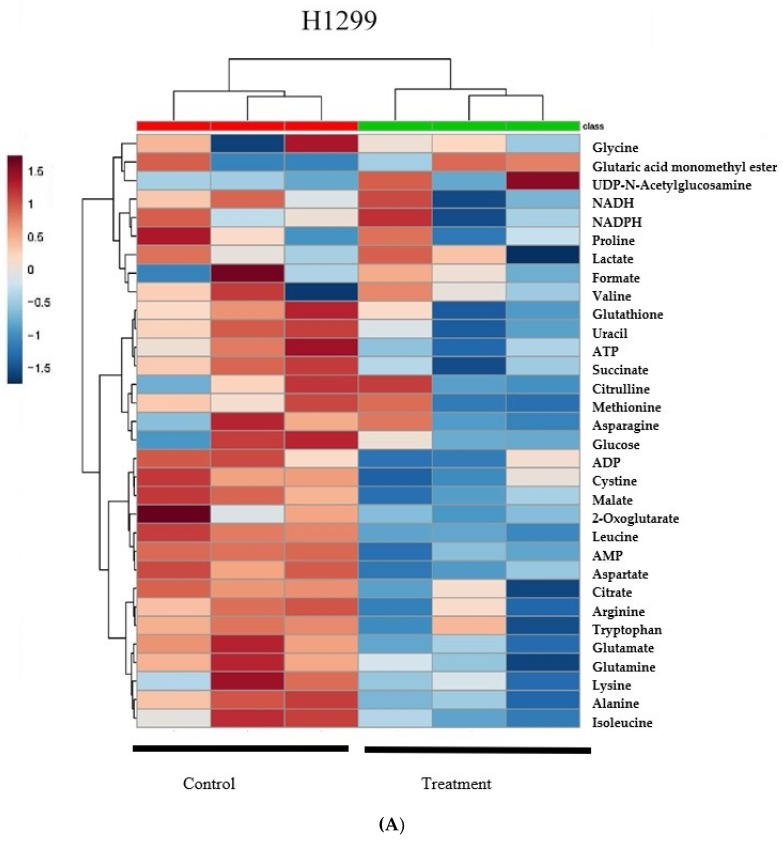

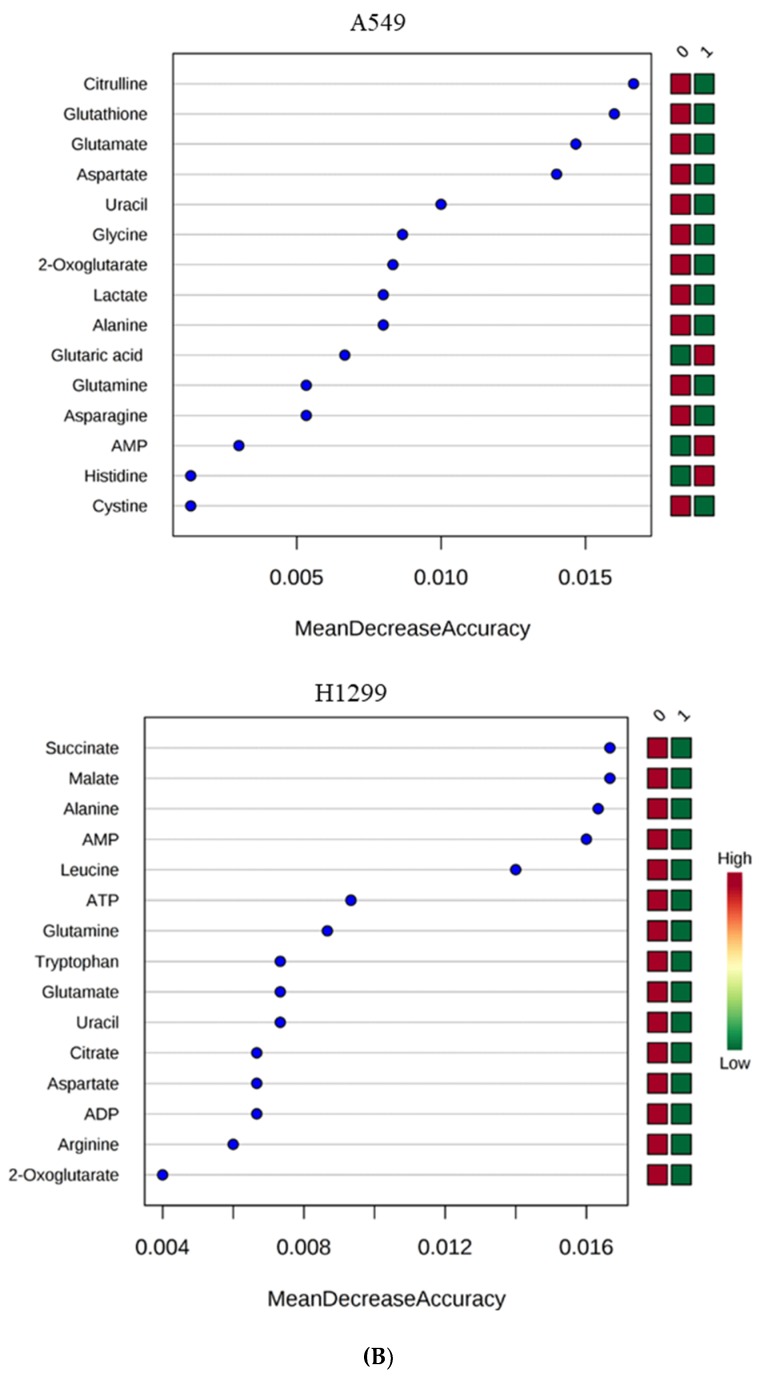
Hierarchical clustering analysis of δT-altered metabolites (Heatmap) and contribution of metabolites in A549 and H1299. The metabolites, quantified with Chenomx software analysis of NMR spectra of A549 and H1299 cells after incubating with or without δT for 72 h, were used to generate the heat map (**A**) using Metaboanalyst software. Each column represents a sample, and each row represents the expression profile of metabolites. Blue color represents a decrease, and red color an increase. The very top row with green color indicates the control samples and red color row indicates the samples with the 30 µM treatment of δT. Random Forest (**B**) showed in bottom graphs identifies the significant features. The features are ranked by the mean decrease in classification accuracy when they are permuted.

Heatmap analysis from MetaboAnalyst 3.0 revealed that A549 and H1299 cell lysates had similar changing trends in metabolites of δT treated groups versus control (Figure 2A), which suggests that the supplement of δT impacts both cell lines in a similar manner. At the same time, our heatmap results also revealed that control and treatment groups supplemented with δT were clustered into two major groups (Green and Red groups at the top of the Heatmap) which suggest clear separation in two groups with their metabolites and also validates the separation in OPLS-DA analysis. The random forest importance plot identified 15 metabolites key in classifying the data with aspartame, alanine, leucine, glutamate glutathione, and glutamine having the most influence on classification (Figure 2B).

**Figure 3:**
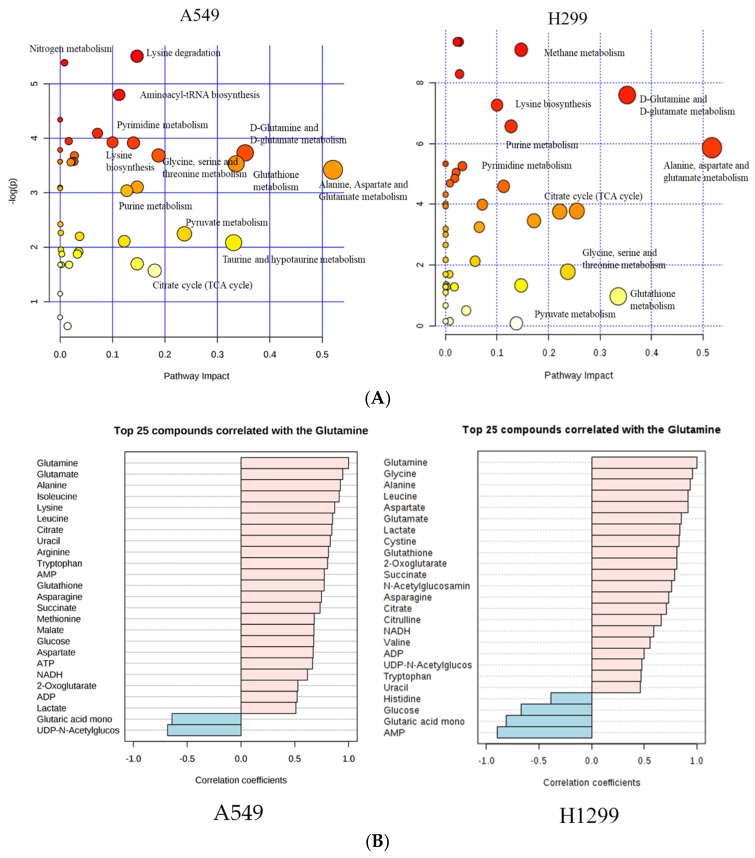
The most predominant altered metabolic pathways (**A**) and top 25 metabolites correlated with glutamine (**B**). Summary of the altered metabolism pathways (**A**) after treating with/without δT for 72 h, as analyzed using MetaboAnalyst 3.0. The size and color of each circle was based on pathway impact value and *p*-value, respectively. Circles, larger and higher along the *Y* axis, show higher impact of pathway on the organism. The top 25 metabolites, correlating with glutamine level (**B**) after treating with/without δT for 72 h. *X*-axis shows maximum correlation; pink color shows positive correlation whereas blue shows negative correlation.

To further comprehend the biological relevance of the identified metabolites from Chenomx analysis, we performed pathway analysis using MetaboAnalyst 3.0 software [25]. Some of the key altered pathways identified from pathway analysis include lysine biosynthesis, purine metabolism, alanine, aspartate and glutamate metabolism, glutamine and glutamate metabolism, citrate cycle (TCA cycle), and pyruvate metabolism for both cell lines (Figure 3A).

As random forest importance plot and pathway analysis indicate that glutamine-based metabolites play a significant contribution to glutamine metabolism and related pathways, correlation between other metabolites were assessed using Pearson correlation analysis to validate the relationship between glutamine and metabolites in other pathways. Interestingly, nearly 20 metabolites showed more than (>0.7) correlation with glutamine and metabolites belonging to the key impaired pathways identified from pathway analysis using MetaboAnalyst 3.0 software. The metabolites in glutamine and glutamate metabolism include glutathione, glutamate, 2-oxoglutarate which show a 0.9, 0.7, and 0.6 correlation in A549 and 0.8, 0.8, and 0.8 correlation in H1299 (Figure 3B).

### 2.3. δT Inhibits Glutamine Transporters (LAT-1 and ASCT2) and the mTOR Pathway in A549 and H1299 Cells

**Figure 4:**
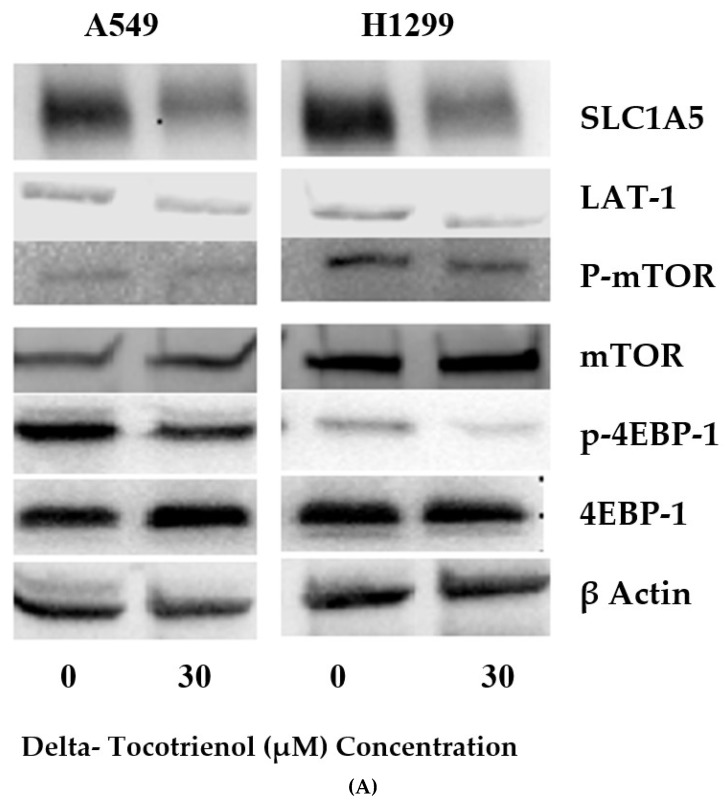

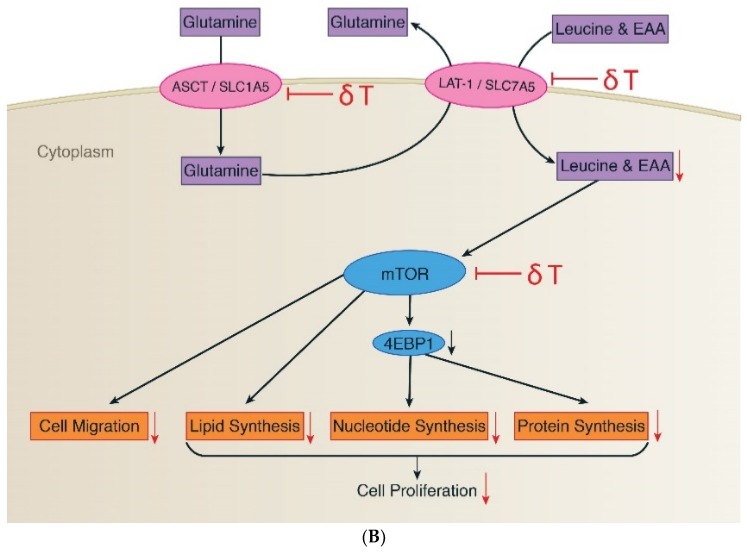
δT inhibits glutamine transporters (LAT-1 and ASCT2) and the mTOR pathway in A549 and H1299 cells. (**A**) The expressions of LAT-1, ASCT2, p-mTOR, mTOR, p-4EBP-1, 4EBP1, and β-actin proteins were detected by Western blot analysis in A549 and H1299 after treating with 0 µM and 30 µM concentrations of δT for 72 h. (**B**) The fate of glutamine uptake in A549 and H1299 involving metabolites (purple), associated key proteins (pink), and functions (orange). Glutamine in cancer facilitates exchanging of EAAs (essential amino acids) into proliferating cells via glutamine transporters (LAT1 and ASCT2), which induces mTOR activation in A549 and H1299. Activated mTOR then promotes protein translation and cell growth via activation of its downstream genes 4EBP1. The black arrows indicate pathway direction, while the red downward arrows indicate inhibition.

Metabolomic analysis and subsequent quantification of metabolites using Chenomx NMR suite (Edmonton, AB, Canada) revealed the potent effect of δT on glutamine metabolism, downstream metabolites of glutamine and essential amino acids (Figure 1 and Figure 2, Table 1). Current literature provides evidence that glutamine uptake and some essential amino acids, including leucine, are associated with the activation of the mTOR pathway [37]. Thus, Western blot analysis was performed to investigate the effect of δT on the mTOR pathway and glutamine transporters. Upon intervention with δT (30 µM), the glutamine transporters (LAT-1 and ASCT2) and key mTOR pathway proteins (P-mTOR and p-4EBP-1) were found to be inhibited, relative to the untreated controls (Figure 4).

## 3. Discussion

In this study, we used multivariate analysis of NMR spectra and NMR quantification data to observe differences in the intracellular metabolomes. We discovered clear differences in the intracellular metabolomes, and subsequently the contributing metabolites, of the control and δT treated cells using OPLS-DA and Heat map analysis (Figure 1 and Figure 2A). Also, we observed a minor difference in the results obtained through multivariate analysis of NMR spectra and NMR quantification variation in this analysis which is common in metabolomic data sets. This type of variation is well documented in several publications in the current literature [6]. Most variations arise from the metabolites present in very low concentrations. In addition, metabolites whose resonances yield a very high number of overlapping peaks also suffer from variations in quantitation [6]. The two different methods were therefore used in conjunction to verify the data.

Previously, using histone ELISA and ANNEXIN V stain-based flow cytometry analysis, we reported that the 10 to 30 μM range of δT was not necrotic to A549 and H1299 cells, and that it induced apoptosis in a dose-dependent manner [11,12]. Also, using MTS and clonogenic assays in the previous studies, we demonstrated that 30 μM of δT inhibited cell growth significantly in the A549 and H1299 cells lines [12]. Other metabolomics investigations have also reported changes in metabolism after inducing apoptosis in different cancer types, namely leukemia cell lines [38]. Our data suggests that metabolite changes in the control vs. δT treated lung cancer cell populations are a result of induction of apoptosis after δT treatment. 

The role of natural dietary components in cancer growth and progression has become a very popular subject with minimum effect or no effect on normal cells. Several cell culture studies showed that δT was not causing apparent impairment towards the noncancerous cell lines, although it significantly effects different cancer cell types, including lung cancer. For instance, Human Fetal Lung Fibroblast Cells treated with 100 µm or higher of δT did not show any toxic effect including induction of apoptosis and DNA damage [18]. In another study, 10 μM DT3, a lower dose than our treatment, was determined to be nontoxic, and enhanced cell viability and proliferative potential in the human lung fibroblast cell lines MRC-5 and HFL1, as shown by WST-1 and clonogenic assays [39]. In addition, Immortal human pancreatic duct epithelial cell lines did not show any significant inhibitory effect on cell proliferation and cell cycle progression when they were incubated with δT [40]. Similarly, normal human melanocytes treated with δT (5–20 μg/mL) for 24 h or 48 h did not affect cell growth at both time intervals [41]. Preclinical and clinical evidence also supports the use of δT to reduce tumor growth with no effects on healthy humans or animals, making δT attractive compounds. No adverse effects were observed upon administration of 300 mg/kg dose of δT, in any tissues or organs of mice [42]. In humans, δT can be safely administered at doses up to 1600 mg twice daily [43]. In another study with osteopenic women, supplementation for 12 weeks did not affect body composition, physical activity, quality of life, or intake of macro- and micronutrients [44]. All of the aforementioned studies used δT concentrations above 30 μM that we used for this study, and it is obvious that δT does not affect healthy cells including human fetal lung fibroblast cells. Therefore, a control arm of normal lung cells with expressed or unexpressed LAT1 and/or ASCT2 were not included in our study design. 

Further, LAT1 or ASCT2 transporters with cancer is nowadays well-assessed [9]. Overexpression of LAT1 is well described in many human cancers and it certainly relates to metabolic changes occurring in cancer development and progression [45]. LAT-1 is expressed in cancers of most human tissues according to GENT database [46], which suggests an important role of LAT-1 expression on cancer development. In contrast, it is poorly expressed or, in some cases, absent in most of the corresponding noncancer human tissues [46]. In the immunohistochemistry analysis of the normal lung, LAT1 protein was identified only on granular regions in the cytoplasm of chondrocytes of the bronchial cartilage, serous cells of the bronchial glands, and alveolar macrophages within the normal lung, whereas the expression was zero for nonciliated bronchiolar epithelial cells (Clara cells), goblet cells of the bronchus, mucinous cells of the bronchial glands, and alveolar type I or type II cells [47]. In the same study, expression of LAT1 protein appeared in the cytoplasm of bronchial surface epithelial cells as a single nodular spot, which was considered to represent an intracellularly localized nonfunctional protein [47]. ASCT2 transporters also are poorly expressed or, in some cases, absent in most of the corresponding noncancer human tissues according to GENT database [46]. Hassanein et al. identified ASCT2 transporters expressed in stage I NSCLC when compared to matched controls using shotgun proteomic analysis [48]. In addition, ASCT2 deficient mice showed regular functions such as normal B-cell development, proliferation, and antibody production [49]. Therefore, control arms of normal lung cells that are expressed or unexpressed (LAT1 and ASCT2) was also not included in our study design as there was a minimum expression and/or functionality observed for LAT1 and ASCT2 in other tissues and noncancerous tissues. 

A significant reduction of glutamine, glutamate, GSH and 2-oxoglutarate after treating with 30 µM of δT on NSCLC cell lines was observed (Table 1). The key aberrant pathways identified using the pathway analysis tool include glutamate and glutamine, alanine, aspartate, glutathione metabolism, and the TCA cycle (Figure 3). In addition, the metabolites identified from these pathways show a strong correlation with glutamine levels (Figure 3B). Further, glutamine and its related metabolites were identified in the S-plot of OPLS-DA analysis and the Random Forest importance plot as the key players causing the separation, reflecting the differences in their metabolomic profiles (Figure 1 and Figure 2B). Glutamine deprivation has been shown to induce apoptosis in hepatoma, hybridoma, leukemia, myeloma, and fibroblast cells [50]. In contrast, increased levels of glutamine were detected in lung cancer tissue especially in NSCLC when compared to other types of cancer, such as colon or stomach cancer [47]. Glutamine dependency has been reported in H1299 and A549 cells [28]. Our findings strongly suggest the beneficial impact of δT on glutamine and related pathways in non-small cell lung cancer cells. 

**Figure 5:**
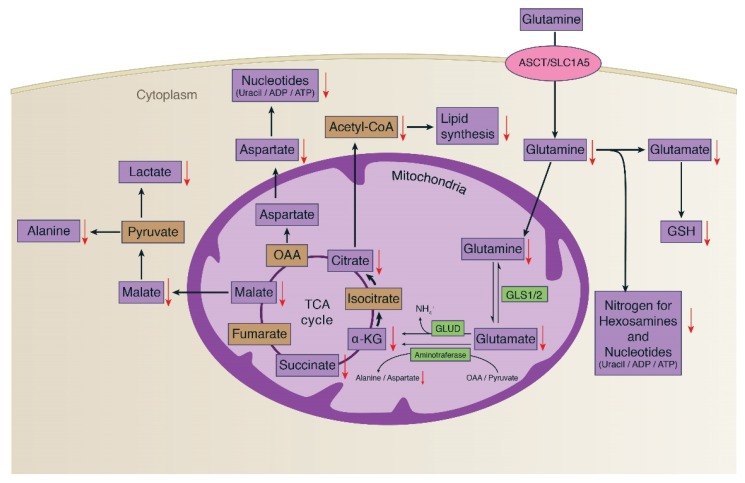
Glutamine metabolism and the effect of δT on glutamine metabolism in A549 and H1299 cells. Glutamine mainly replenishes the TCA cycle intermediates and GSH synthesis in cancer cell proliferation. In the process, glutaminase enzymes (GLS1/2) catalyzes the conversion of glutamine to glutamic acid and the subsequent conversion of glutamate to α-ketoglutarate (α-kG), catalyzed by glutamate dehydrogenase (GLUD) and amino transferase. This process supports for uncontrolled cell proliferation in cancer cells and requires a large number of macromolecules to create new biomass, including DNA, proteins, and lipids. The black arrows indicate the pathway’s direction, while the red downward arrows indicate the inhibition of metabolites as an effect of δT treatment.

Considering metabolism of glutamine (Figure 5), one of its major roles in cancer cell proliferation is to replenish the TCA cycle intermediates removed by the process called glutaminolysis, and GSH synthesis [30,31]. In the process of glutaminolysis, the glutaminase enzyme (GLS1/2) catalyzes the conversion of glutamine to glutamic acid and the subsequent conversion of glutamate to α-ketoglutarate (2-oxoglutarate), catalyzed by glutamate dehydrogenase (GLUD) [32]. Aminotransferase also catalyzes the reaction from glutamate and oxaloacetate to aspartate or alanine and α-ketoglutarate. In this study, a significant reduction of glutamine, glutamate, and TCA cycle intermediates after treating with 30 µM of δT was observed, which is an indicator of reduced energy metabolism (Figure 5). In cancer cells, the enhanced production of 2-oxoglutarate and glutamate from glutamine metabolism can be observed, as it helps to maintain the citric acid cycle intermediate for energy production [32]. Glucose and glutamine provide substrates for macromolecular synthesis supplying both ATP and carbon skeletons in cancer cells [29]. This supports uncontrolled cell proliferation in cancer cells and requires a large number of macromolecules to create new biomass, including DNA, proteins, and lipids [28]. Our data suggests that by decreasing the availability of glutamine, δT retards this process, thereby leading to inhibition of uncontrolled cell proliferation in A549 and H1299 as reported in our previous studies [11,12,35]. 

Considering possible causes for the significant decrease in glutamine and its downstream metabolites, we hypothesized that it may be due to inhibition of glutamine transporters. We thus measured the protein levels of glutamine transporters, namely LAT1 and ASCT2, known to play a fundamental role in glutamine uptake process in normal cell physiology. LAT-1 facilitates glutamine efflux in exchange for the influx of leucine and other essential amino acids (EAA) across the cell membrane; similarly, ASCT2 mediates uptake of neutral amino acids including glutamine [51]. Our observations from western blot analysis established that δT treatments inhibit the expression of LAT-1 and ASCT2 (Figure 4). We also quantified detectable EAA including leucine in cell lysates, the concentration of which were decreased significantly after treating NSCLC cells with δT by NMR analysis. Inhibition of EAA in A549 and H1299 cells upon δT treatment reflects function of LAT-1 which facilitate glutamine efflux in exchange for the influx of leucine and other essential amino acids (EAA). This supports the beneficial effects of δT on LAT1 transporters inside A549 and H1299 cells. In addition to facilitating the transport of EAAs for protein synthesis, LAT1 and ASCT2 stimulate the growth of cancer cells via mTOR [27,52,53]. In head and neck squamous cell carcinoma cell lines, inhibition of the LAT-1 transporter using an inhibitor lowered the levels of phosphorylation of mTOR and its downstream signaling molecules [54]. Thus, if the inhibition of glutamine transporters and EAA uptake with δT treatment is valid, it is logical to expect inhibition or lower activation of mTOR pathway after treating with δT in NSCLC. Indeed, we observed lower activation of mTOR along with LAT-1 and ASCT2 after treating with δT, using Western blot analysis, which illustrates that inhibition of glutamine transporters affect the mTOR signaling pathway (Figure 4).

mTOR functions are mediated by two downstream proteins, the eukaryotic initiation factor 4E (eIF4E)-binding protein 1 (4E-BP1) and p70 ribosomal S6 kinase 1 (p70S6K1, S6K1) (Figure 4) [55]. For further confirmation, we tested the expression levels of downstream genes of mTOR namely P-4E-BP1. We observed the similar inhibitory effect on mTOR downstream proteins 4E-BP1suggesting an inhibitory effect of glutamine transporters passing through mTOR to downstream pathway (Figure 4). mTOR downstream proteins 4E-BP1 and S6K1 regulate F-actin reorganization, focal adhesion formation, and tissue remodeling through the proteolytic digestion of extracellular matrix via upregulation of matrix metalloproteinase 9 (MMP-9) [56]. Interestingly, in our previous study, we observed that δT reduced cell migration, invasion and adhesion in a dose- and time-dependent manner, and inhibited MMP-9 expressions in NSCLC cells [13,34], which is an additional supporting inhibitory function of δT. 

Further, in the previous study, we demonstrated that δT induces apoptosis in a dose-dependent manner in NSCLC from Annexin based flow cytometry analysis and histone ELISA [12]. The current literature also provides evidence to support the relationship between GSH and apoptosis. For instance, GSH depletion in cancer cells induces apoptosis in vitro and in vivo [57]. Dalton TP et al. showed GSH-depleted knockout mouse of *γ*-GCS died from massive apoptotic cell death [58]. Elevated levels of GSH are also associated with apoptotic resistant phenotypes in several models of apoptosis in previously reported studies [59,60], and GSH depletion by itself has been observed to either induce or stimulate apoptosis [59,61]. GSH quantification, after treating with δT in A549 and H1299 cells, shows a clear decline in intercellular GSH levels in both cell lines (Table 1). The results reveal there may also be a possible association between GSH levels and induction of apoptosis in NSCLC cells after treating with δT.

## 4. Materials and Methods 

### 4.1. Cell Culture and Treatment with δ-T

NSCLC cell lines A549 and H1299 were cultured in RPMI medium (Mediatech, Manassas, VA, USA) supplemented with 10% fetal bovine serum and 1% penicillin and streptomycin in 5% CO_2_ at 37 °C. The culture medium was renewed every 2 to 3 days. Adherent cells were detached by incubation with trypsin-EDTA and centrifuged at 80× *g*. The treatment media was prepared by mixing δT (<0.01% DMSO as a vector) in the RPMI medium, whereas the control was treated only with RPMI media. Three δT solutions at concentrations of 10 μM, 20 μM, and 30 μM containing <0.01% DMSO were chosen as the treatment concentration based on our previous studies. δT was a gift from the American River Nutrition for this study. 

### 4.2. Intracellular Metabolite Extraction and Determination

We used a modified method which is explained in Saadat et al., 2018 [62]. In brief A549 and H1299 lines were seeded at a density of 2 × 10^6^ per 100-mm dish for 24 h, followed by replacement of media absent or supplemented with different δT concentrations (10 µM, 20 µM, and 30 µM) at 37 °C. Cells were then incubated for another 72 h before extracting metabolites. Before extracting intracellular metabolites, existing culture media was removed on ice followed by washing twice with ice-cold PBS. Two milliliters of ice-cold methanol was added while scraping with cell scrapers on ice. The Petri dish was shaken for 5 min at 4 °C and ice-cold methanol was transferred into Eppendorf tubes. The cell debris was removed by centrifugation and all the extraction solvents were readily removed before NMR analysis by a Speed Vac at room temperature. Subsequently, the intracellular metabolites powder was prepared by evaporating with methanol, and redissolving in 450 μL D_2_O containing 0.5 μM 2,2-Dimethyl-2-silapentane-5-sulfonic acid (DSS) as a spectral calibration standard and 10 μM imidazole as a pH indicator. An additional Petri dish was prepared for each treatment/control with the same conditions and cells collected from the additional petri dish were used for analyzing total protein. The total protein quantifications include control-A549 (1.283 mg), 30 µM-A549 (1.099 mg), control-H1299 (1.325 mg), and 30 µM-H1299 (1.276 mg). The intracellular metabolite powder was redissolved in D_2_O and normalized based on the total protein contained in additional petri with corresponding treatment before performing NMR. We made sure to maintain the final concentration of internal standards at aforementioned levels. 

### 4.3. 1H-NMR Spectroscopy

High-resolution 1H-NMR spectra of intracellular metabolites were obtained on a Varian 600 spectrometer operating at 600 MHz after normalizing the samples by total protein concentrations using BCA Protein Assays (Thermos Fisher Scientific, Rockford, IL, USA). 1H-NMR spectra of intracellular extracts were acquired using a 6-kHz spectral width and 64 K data points. The acquisition time was 5.44 s and the relaxation delay was 14.56 s with 64 scans. 

### 4.4. 1H-NMR Spectroscopy Processing 

After NMR analysis, Free Induction Decay (FID) files were obtained and processed using NMR processing software ACD (Advanced Chemistry Development, Inc. Toronto, ON, Canada). NMR spectra of all the samples were stacked and processed simultaneously. First, FID files were Fourier-transformed to visualize spectra followed by phasing, baseline correction and binning with the auto option of the software. After completing these steps, the full spectra, as a batch, were divided into 1000 bins using the intelligent bucketing algorithm in ACD software, giving a numerical value for corresponding peaks, and converted into a data table. Intelligent bucketing in ACD is an algorithm that was designed to make decisions as to where a bucket division should be. Intelligent bucketing chooses integral divisions based on local minima and therefore avoids the reduction of data resolution, while aligning the spectra as a batch. 

### 4.5. Quality Control

Relative standard deviation (RSD) values were calculated for each treatment group separately and Technical variation within metabolomics datasets, recorded using one dimensional NMR maintained less than <8% (reported as the median spectral RSD)

### 4.6. Multivariate Data Analysis: OPLS-DA

The processed, digitized NMR spectral data table from ACD software (version 10) was imported into the SIMCA (version 15) software (Sartorius Stadium Biotech, Germany for Multivariate data analysis (MVDA). The data table was transposed and labeled accordingly. The integrals corresponding to the spectral region from 4.5 to 6 ppm were excluded as this region contains water peaks and exchangeable protons. Spectral regions displaying no peaks, DMSO, and spectral regions of methanol to all the samples were also excluded from the dataset. PCA, OPLS-DA models were created by generating optimum number of principal components needed to fit the data, using the autofit option in the software. Each model’s characteristics are described by how well it fits the data and its ability to predict new data accurately. Thus the value for R2 describes how well the data fits the model while the value of Q2 relates to the models ability to predict unknown data correctly. These are calculated by the for the purpose of evaluating and validating the models generated. The following cutoff criteria are used for validating the models that were generated. For NMR metabolomic data, it is recommended that the model generated has a Q2 > 0.5, a value of R2 higher than Q2 with the difference between them being no greater than 0.3. These criteria were adhered to for all the models utilized for the investigation. Samples were identified and distinguished by their respective labels and colored for visual convenience. The data was subjected to Pareto-scaling prior to analysis. The Hotelling T^2^ test (based on the 95% confidence interval) and DMOD-X test (based on the distance from the model plane) was used to remove any statistically extreme outliers while maintaining a minimum of 4 replicates in each group. Initially, unsupervised Principal Component Analysis (PCA) was performed to view the clustering effects in the samples (Supplemental Materials). Subsequently, OPLS-DA, a supervised pattern recognition method, was performed to maximize the identification of variation between groups tested. 

### 4.7. Metabolite Identification and Quantification from Chenomx NMR Suite

The metabolites were identified using Chenomx NMR suite (Chenomx Inc., Edmonton, AB, Canada). The fid files from the 1D 1H-NMR spectra were imported into the Chenomx software. This software has its own processing interface where spectra were Fourier-transformed and baseline corrected. Phasing was done using DSS reference peak at 0.0 ppm, and the water peak was also deleted. The processed spectra were analyzed in the profiler module of the software. The 600 MHz library with the corresponding pH was selected. Identification and concentrations of different metabolites were calculated by fitting the set of peaks for those compounds in the sample spectrum. If the area was crowded with many peaks, then multiple metabolites were adjusted at one time to match the reference spectrum closest to the sample spectrum. The identified and quantified compounds were then exported into an excel sheet.

### 4.8. Additional Multivariate Data Analysis and Metabolic Pathway Identification Using MetaboAnalyst 3.0 Software

MetaboAnalyst 3.0 software, a web-based metabolomics data processing tool [63], was used to statistically analyze the metabolites identified using Chenomx NMR suite. Quantified data from Chenomx NMR suite were scaled using range scaling algorithm. Clustering differences, heat maps, and a Random Forest analysis plot were generated. Further, the top 25 metabolites correlating with glutamine were identified using Pearson correlation analysis and the significant features were identified by Random Forest analysis. Additionally, quantified data from Chenomx NMR suite was transferred into an excel table which allowed us to perform a Student’s *t*-test and calculate fold changes. A *p*-value of less than 0.05 was considered to be statistically significant for univariate analysis.

Metabolic pathway identification was performed with the pathway analysis option of Metaboanalyst 3.0 software. Briefly, the Homo Sapiens Pathway Library was selected as a reference, and the pathway analysis was performed to generate pathway analysis output on all matched pathways, based on the *p*-values from pathway enrichment analysis and pathway impact values from pathway topology analysis. 

Further, metabolites that were changing most significantly between the control and 30 µM treatment were traced back to their origin, and the pathways were interpreted for metabolism changes using current biochemistry.

### 4.9. Western Blot for Protein Expression Analysis

One million cells of each of A549 and H1299 were seeded in 100-mm dishes and incubated for 24 h; then, the original media was replaced by media with/without δT and incubated for another 72 h. After 72 h incubation, cells were washed with ice-cold PBS and lysed in the cold 1X cell lysis buffer (Cell Signaling Technology, Danvers, MA, USA) for 30 min on ice with 1X protease inhibitor (Cell Signaling Technology, Danvers, MA, USA). The cell lysate was kept at −80 °C overnight before quantifying. 

Protein concentrations were estimated using Pierce BCA Protein Assay kit (Bio-Rad Laboratories, Hercules, CA, USA). Total cell lysates (40 µg) were mixed with equal amounts of 6x laemmli buffer (Bio-Rad Laboratories, Hercules, CA, USA), followed by boiling at 100 °C for 5 min. Samples were loaded on 10% SDS-polyacrylamide gel electrophoresis, and then the gel was electrophoretically transferred to a nitrocellulose membrane (Whatman, Clifton, NJ, USA) in transfer buffer (25 mM Tris, 190 mM glycine, 20% methanol) using a Bio-Rad Trans-Blot^®^ Turbo™ Transfer System (Hercules, CA, USA). The membranes were incubated for 1 h at room temperature with 5% BSA in 1x TBS buffer containing 0.1% Tween. After incubation, the membranes were incubated overnight at 4 °C with primary antibodies (1:1000). The following antibodies ASCT2, LAT-1, p-mTOR, mTOR, p-4EBP-1,4-EBP1, and B-actin (Cell Signaling Technology, Danvers, MA, USA) were used in the analysis. The membranes were washed three times with TBS-T and subsequently incubated with the secondary antibodies (1:5000) containing 2% BSA for 2 h at room temperature. The signal intensity was then measured by chemiluminescent imaging with ChemiDoc XRS (Bio-Rad Laboratories, Hercules, CA, USA).

## 5. Conclusions

In this work, the anticancer effects of δT on NSCLC cell lines A549 and H1229 were investigated and confirmed by 1H-NMR metabolomics analysis. A closer look into the intracellular metabolome of NSCLC cells revealed significant and potentially beneficial alterations in glutamine concentrations and related metabolism upon treatment with δT. The data purports that δT exerts its action by inhibiting glutamine uptake into proliferating cells by inhibition of glutamine transporters, thereby resulting in inhibition of cell proliferation and induction of apoptosis via downregulation of the mTOR pathway (Figure 4B and Figure 5). Through this work, NMR-based cellular metabolomics helps provide possible opportunities for evaluating the therapeutic effect of phytochemicals and systemic changes in cancer metabolism.

## Figures and Tables

**Table:** (A)

**Metabolite Name**	**Mean ± SD (Control)**	**Mean ± SD (Treatment)**	***p*-Value**	**Fold Changes Control/Trt**
***Amino Acids***				
Aspartate	102.3 ± 11.9	55.9 ± 4.7	0.0016	1.8
Glutamate	80.8 ± 7.9	48.7 ± 4.7	0.0019	1.7
Leucine	33.7 ± 4.1	17 ± 3.7	0.0030	2.0
Glycine	33.1 ± 1.2	20.4 ± 4.2	0.0035	1.6
Alanine	31 ± 1.4	19.8 ± 3.9	0.0045	1.6
Glutamine	99.9 ± 6.7	64.7 ± 13.3	0.0073	1.5
Histidine	54 ± 8.4	85.9 ± 31.3	0.0815	0.6
Asparagine	116.9 ± 16.2	54.5 ± 13.1	0.0033	2.1
Taurine	90.3 ± 19.9	78.2 ± 26.8	0.2822	1.2
Valine	23.8 ± 1.4	21.6 ± 6.3	0.2878	1.1
Tryptophan	81.3 ± 15	72.7 ± 28.7	0.3340	1.1
Proline	51.9 ± 49.3	63.7 ± 25.7	0.3659	0.8
Lysine	41.6 ± 22.8	37.2 ± 6.1	0.4075	1.1
Isoleucine	31.5 ± 9.9	30.6 ± 7	0.4499	1.0
Methionine	5.8 ± 5.3	5.5 ± 3.4	0.4653	1.1
Arginine	nd	nd		
***Intermediate of TCA Cycle and Energy Metabolism***				
Lactate	138.5 ± 5.6	99.9 ± 3.6	0.0003	1.4
2-Oxoglutarate	43.6 ± 3.3	29.3 ± 4.7	0.0061	1.5
AMP	32.1 ± 5	45 ± 1.7	0.0063	0.7
Glutaric acid monomethyl ester	17.8 ± 6.4	34 ± 2.8	0.0077	0.5
Malate	90.2 ± 10.7	48.7 ± 10.3	0.0111	1.9
Succinate	9.3 ± 2.6	5.2 ± 2.8	0.0645	1.8
Glucose	119.1 ± 53.4	187.3 ± 63.7	0.1139	0.6
ADP	47.8 ± 8.3	40.8 ± 4.8	0.1370	1.2
Citrate	42.4 ± 3.8	35.6 ± 11.6	0.1959	1.2
NADH	38.4 ± 3.5	43.4 ± 16	0.3040	0.9
NADPH	47 ± 6.3	51.3 ± 12.5	0.3118	0.9
ATP	42.2 ± 5.4	42.9 ± 11.3	0.4653	1.0
***Nucleic acid Associataed Metabolites***				
Uracil	98 ± 14.1	60.1 ± 24	0.0387	1.6
UDP-N-Acetylglucosamine	6.9 ± 2.1	3.9 ± 3.4	0.1266	1.8
***Other***				
Glutathione	69.6 ± 2.1	41.7 ± 6.7	0.0011	1.7
Citrulline	81.9 ± 5.1	63.9 ± 13	0.0438	1.3
Cystine	81.4 ± 6.3	58.4 ± 19	0.0582	1.4
N-Acetylglucosamine	21.9 ± 9.3	12.8 ± 5.2	0.1065	1.7
Formate	294.3 ± 68.5	312.8 ± 8.9	0.3334	0.9
Fumarate	25 ± 3.2	27.7 ± 5	0.2363	0.9

**Table:** (B)

**Metabolite Name**	**Mean ± SD (Control)**	**Mean ± SD (Treatment)**	***p*-Value**	**Fold Changes Control/Trt**
***Amino Acids***				
Aspartate	105.5 ± 3.5	77.4 ± 4.3	0.0010	1.4
Glutamate	80.1 ± 5.7	49.3 ± 6.2	0.0033	1.6
Leucine	31.8 ± 1.3	18.3 ± 0.8	<0.0001	1.7
Glycine	28.2 ± 4.7	18.1 ± 3.2	0.0561	1.6
Alanine	28.8 ± 2.2	18.2 ± 2.3	0.0044	1.6
Glutamine	75.3 ± 5.1	53.7 ± 8.4	0.0177	1.4
Histidine	ND	ND		
Asparagine	105 ± 21	84 ± 23.3	0.1986	1.3
Taurine	ND	ND		
Valine	28.8 ± 4.9	21.7 ± 5.6	0.1706	1.3
Tryptophan	36.8 ± 2	17.8 ± 11.4	0.0401	2.1
Proline	90.2 ± 39.3	74.3 ± 34.9	0.3453	1.2
Lysine	38.8 ± 11.3	19.4 ± 7.1	0.0547	2
Isoleucine	37.2 ± 4.9	23.8 ± 2.7	0.0138	1.6
Methionine	8.7 ± 0.8	6.7 ± 1.9	0.1247	1.3
Arginine	43.8 ± 2.7	28.4 ± 6.6	0.0189	1.5
***Intermediate of TCA Cycle and Energy Metabolism***				
Lactate	125.8 ± 7.3	122 ± 15.4	0.3857	1
2-Oxoglutarate	32.5 ± 7.9	17.2 ± 1.5	0.0272	1.9
AMP	27.5 ± 0.2	13.7 ± 2	0.0003	2
Glutaric acid monomethyl ester	27.4 ± 0	20.6 ± 7.4		1.3
Malate	130.9 ± 7.8	84.7 ± 9	0.0027	1.5
Succinate	13.9 ± 1.7	5.3 ± 3.8	0.0215	2.6
Glucose	196.4 ± 50.1	147.1 ± 19.4	0.1324	1.3
ADP	33.6 ± 5.1	14.9 ± 7.7	0.0227	2.3
Citrate	35.2 ± 0.8	25.6 ± 4.3	0.0183	1.4
NADH	65.3 ± 11.7	43.7 ± 30.7	0.2024	1.5
NADPH	48.6 ± 11.1	38.1 ± 23.5	0.2996	1.3
ATP	43.5 ± 7.8	22.2 ± 5.5	0.0171	2
***Nucleic acid Associated Metabolites***				
Uracil	88.5 ± 11.9	40.2 ± 16.3	0.0139	2.2
UDP-N-Acetylglucosamine	ND			
***Other***				
Glutathione	42.3 ± 4.5	28 ± 6.5	0.0319	1.5
Citrulline	65.4 ± 20.6	53.4 ± 25.4	0.3156	1.2
Cystine	61 ± 7.2	26.3 ± 14.1	0.0338	2.3
N-Acetylglucosamine				
Fumarate				
Formate	354.5 ± 90.9	346.7 ± 41	0.4585	1
Tyrosine	12.9 ± 0.6	67.8 ± 9.1	0.0134	0.2

## Supplementary Materials

The following are available online at https://www.mdpi.com/2218-1989/9/3/50/s1. Table S1: List of metabolite concentrations determined using Chenomx NMR Suite in A549 cells. Table S2: List of metabolite concentrations determined using Chenomx NMR Suite in H1299 cells. Figure S1: Effects of δT on A549 (A) and H1299 (B) on the metabolome of lung cancer cell lines.
