# Supplementary material for: Delta-Tocotrienol Modulates Glutamine Dependence by Inhibiting ASCT2 and LAT1 Transporters in Non-Small Cell Lung Cancer (NSCLC) Cells: A Metabolomic Approach
Source: Metabolites. 2019 Mar 13;9(3):50. doi: 10.3390/metabo9030050 (PMC6468853; doi:10.3390/metabo9030050)
Supplement: Supplementary file 1 [file metabolites-09-00050-s001.pdf]

Supplement Data:

Delta-tocotrienol modulates glutamine dependence by inhibiting ASCT2 and LAT1 Transporters in non-small cell lung cancer (NSCLC) cells: Metabolomic Approach

Lichchavi Dhananjaya Rajasinghe[1], Melanie Hutchings [1], Smiti Vaid Gupta[1*]
[1] **Department of Nutrition and Food Science, Wayne State University, Detroit, MI, U.S.A;**

Table S1: List of metabolite concentrations determined using Chenomx NMR Suite in A549 cells. All ^{1}H-NMR spectra were processed and analyzed using the Chenomx NMR Suite Professional software (Chenomx Inc., Edmonton, AB), as previously described [1].

**A549 cells**	**Control 1**	**Control 2**	**Control 3**	**Treatment 1**	**Treatment 2**	**Treatment 3**
Metabolite Name	**Concentration (µM)**					
2-Oxoglutarate	40.2	44	46.6	34.7	26.2	27
Acetate	25.4	29.7	28.6	23.2	17.7	26
ADP	42.9	57.3	43.1	40.9	36	45.5
Alanine	30.6	32.5	29.8	17.8	17.3	24.2
AMP	32.5	36.8	27	43.2	46.4	45.3
Asparagine	106.2	135.5	108.9	67.1	55.3	41.1
Aspartate	100.5	91.4	114.9	50.6	58	59.1
ATP	36	45.2	45.4	53.9	31.4	43.3
Citrate	38.3	43	45.7	31.3	26.8	48.7
Citrulline	77.2	87.2	81.3	49	71.4	71.3
Cystine	84	85.8	74.2	44.5	50.7	79.9
Dimethylamine	8	6.2	9.8	403.6	210.8	66.9
Formate	318.9	216.9	347.1	303.7	321.3	313.4
Fumarate	21.6	27.9	25.4	28.3	22.4	32.3
Glucose	180.6	86.5	90	234.7	212.2	114.9
Glutamate	75	89.7	77.5	44.9	47.2	53.9
Glutamine	103.7	92.2	103.8	62.7	52.5	78.7
Glutaric acid monomethyl ester	12.1	24.6	16.5	33.7	31.3	36.8
Glutathione	70.5	67.3	71	36.9	49.3	38.9
Glycine	31.8	33.4	34	22	15.6	23.4
Histidine	46.3	52.9	62.8	56.6	82.2	118.7
Isoleucine	22	30.7	41.7	33.8	22.6	35.2
Lactate	132.3	143	140.2	103.5	99.8	96.4
Leucine	38.2	32.4	30.4	13.4	16.8	20.7
Lysine	66.3	36.9	21.6	32.9	41.5	--
Malate	97.7	82.6	--	49.9	58.2	37.8
Methionine	3.6	11.8	2	5.3	2.2	8.9
N-Acetylglucosamine	31.4	13	21.1	8.2	11.8	18.4
NAD+	43.7	49.3	52.6	23.9	--	53.7
NADH	36.4	42.4	36.3	32.1	--	54.7
NADP+	71.8	29.9	22	6.6	--	44.2

NADPH	40.9	53.3	46.7	50.6	39.1	64
Proline	42	8.2	105.3	38.7	62.2	90
Succinate	8.1	7.5	12.1	2.3	5.5	7.7
Taurine	98.4	104.8	67.6	104.2	79.6	50.8
Tryptophan	64	88.8	91	54.9	57.3	105.7
Tyramine	7.7	22.7	53.9	--	34.1	78.3
UDP-N-Acetylglucosamine	7.2	8.8	4.7	0	5.6	6
Uracil	86.4	113.6	94	80.7	65.8	33.8
Valine	22.4	25	24	24	14.5	26.2

Table S2: List of metabolite concentrations determined using Chenomx NMR Suite in H1299 cells. All ^{1}H-NMR spectra were processed and analyzed using the Chenomx NMR Suite Professional software (Chenomx Inc., Edmonton, AB), as previously described [1].

**H1299 cells**	**Control 1**	**Control 2**	**Control 3**	**Treatment 1**	**Treatment 2**	**Treatment 3**
Metabolite Name	**Concentration (µM)**					
2-Oxoglutarate	23.8	30.8	43	15.1	18.2	18.2
ADP	37.7	26.4	36.6	10	8.9	25.7
Alanine	30	30.7	25.8	20.4	19.2	15.1
AMP	27.2	27.7	27.6	15.9	11	14.3
Arginine	44.9	46.4	40	37.6	24.9	22.7
Asparagine	128.8	108.4	77.8	70.5	116.8	64.7
Aspartate	100.6	107.2	108.8	77	72.4	82.8
ATP	43.9	52.8	33.7	14.6	24.6	27.3
Citrate	34.5	34.7	36.3	31.1	25.1	20.6
Citrulline	65.6	90.5	40.1	36.8	89.3	34.1
Cystine	55.5	56.2	71.2	12.2	--	40.3
Fumarate						
Formate	479.3	319.2	265.1	356.4	391.4	292.3
Glucose	228.5	235.1	125.6	133.9	174.6	132.9
Glutamate	88.2	75.3	76.9	56.5	50.1	41.4
Glutamine	82.4	72.2	71.2	56.5	62.2	42.3
Glutaric acid monomethyl ester	--	--	27.4	26.5	10.1	25.1
Glutathione	41.9	48	37	21.1	36.8	26.2
Glycine	--	32.8	23.5	21	19.6	13.7
Histidine						
Isoleucine	41.1	40.2	30.3	23.4	27.3	20.6
Lactate	124	117.9	135.4	128.7	136.6	100.8
Leucine	31.1	30.7	33.6	19	18.8	17.2
Lysine	50.6	42.3	23.6	27.3	21	10
Malate	132.8	120.5	139.4	84.1	73.9	96

Methionine	7.9	9.8	8.3	5.4	9.4	5.2
N-Acetylglucosamine						
NADH	80.4	51.8	63.7	11.4	84.9	34.8
NADPH	37.3	44.7	63.7	11.5	68.6	34.2
Proline	89.1	42.6	138.8	32.8	118.1	71.9
Succinate	14.5	15.6	11.5	0	8.3	7.6
Taurine						
Tryptophan	39	37.3	34.2	33.4	13.5	6.6
UDP-N-Acetylglucosamine	12.3	--	13.4	--	58.6	76.9
Uracil	94.7	99	71.9	21.2	61.1	38.4
Valine	33.6	--	23.9	20.9	28.9	15.2

A

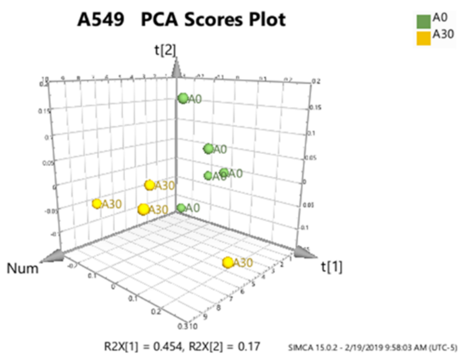

B

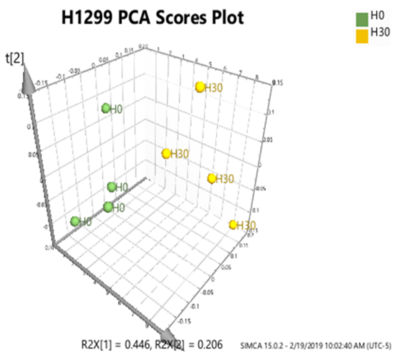

Figure S1: **Effects of δT on A549 (A) and H1299 (B) on the metabolome of lung cancer cell lines.** 3 Dimensional Principal component analysis (PCA) scores plot(A and B), PC1 (t[1]) versus PC2 (t[2]) and numbers showing the unsupervised separation of metabolites profiles among the δT treatments 0(Green) and30 (yellow) after 72 hours incubation. Each symbol represents metabolites from the one Petri dish described at the method section; The ellipses shown in A and B represents the Hotelling's T2 95% confidence interval for the multivariate data. Data is parito scaled.

Reference

1. Wishart DS, Lewis MJ, Morrissey JA, Flegel MD, Jeroncic K, et al. (2008) The human cerebrospinal fluid metabolome. J Chromatogr B 871: 164-173.
